# A newly-recorded species of the genus *Rhodotritoma* Arrow, 1925 (Coleoptera, Erotylidae) from China

**DOI:** 10.3897/BDJ.10.e96740

**Published:** 2022-12-19

**Authors:** Huixin Xu, Jiaxin Pang, Jing Li, Zhiqiang Cheng

**Affiliations:** 1 Hebei Agricultural University, Baoding, China Hebei Agricultural University Baoding China; 2 University of Hawaii at Manoa, Honolulu, United States of America University of Hawaii at Manoa Honolulu United States of America

**Keywords:** pleasing fungus beetle, Erotylinae, Tritomini, taxonomy, key, Oriental Region

## Abstract

**Background:**

The genus *Rhodotritoma* Arrow, 1925 (Coleoptera, Erotylidae, Erotylinae, Tritomini) includes 12 known species worldwide, including three species distributed in China. In the last four decades, no work was conducted on *Rhodotritoma* in China. In this paper, we review the taxonomy of this genus for Chinese fauna and redescribe a newly-recorded species in China.

**New information:**

*Rhodotritomamanipurica* Arrow, 1925 is recorded from China for the first time. The morphological characters of the adult are redescribed in detail and illustrated. A key to species of the genus *Rhodotritoma* Arrow, 1925 in China is provided. Chinese specimens were collected from Tibet Autonomous Region and Yunnan Province, which were then deposited in the Museum of Hebei University. The holotype examined is kept in the Natural History Museum.

## Introduction

The genus *Rhodotritoma* was described by Arrow (1925) for *Triplaxcoccinea* Crotch, 1876, a species distributed in China, India and Burma (Chûjô 1969). *Rhodotritoma* includes 12 species worldwide (Chûjô 1990, Wegrzynowicz 2007) with three species known from China: *R.rubicunda* Araki, 1941 (Taiwan (Araki 1941) and Hainan (Chûjô 1964)); *R.coccinea* Crotch, 1876 (China (Crotch 1876) and Fujian (Mader 1941)) and *R.albofasciata* Nakane, 1981 (Taiwan (Nakane 1981)).

During our examination of specimens from south-western China, a species of new national record, *Rhodotritomamanipurica* Arrow, 1925 from Tibet and Yunnan was found. Before this study, the species was known only from Assam, India (Arrow 1925). Here, based on the comparison of the holotype, we redescribed and illustrated the morphological characters of the adult found in China. A key to the species of *Rhodotritoma* from China is given below.

## Materials and methods

The specimens were softened in hot water for 12 hours. After that, the abdominal segments and genitalia were detached from the body. Male and female genitalia were immersed in boiling sodium hydroxide (NaOH) solution (5%) for five minutes and then cleaned with distilled water. Morphological characters were observed and illustrated with a Nikon SMZ800N stereomicroscope and modified with Adobe Photoshop CS6.0. Habitus photographs were taken with an Olympus E-M5Ⅱ camera. The species has sexual dimorphism. Males can be distinguished by the inner odontoid processes of the pro- and mesofemora. Two males and two females were dissected. Morphological terminology for external structures follows Lawrence (Lawrence et al. 2010, Lawrence et al. 2011).

We examined the holotype specimen deposited in the Natural History Museum (NHML) and compared the specimens from China with the holotype in morphological characteristics.

## Taxon treatments

### Rhodotritoma

Arrow, 1925

F8337C98-F40A-5B59-AC83-7DF5C68F3FB7

Rhodotritoma Arrow, 1925 - Arrow 1925: 115. Type-species: *Triplaxcoccinea* Crotch, 1876.

#### Diagnosis

Parts of the following characters combined the features from Arrow (1925) and Chûjô (1969).

Body oval, dorsally convex in lateral view. Head with a pair of stridulatory files on occipital region, both sides with ridge processes, anterior part of frons shallowly impressed at each side. Clypeus with anterior border feebly emarginate. Antennae slender, antennomere Ⅱ short, antennomere Ⅲ more than twice as long as wide, antennomeres Ⅵ-Ⅷ slender, antennal club composed of last three antennomeres loosely articulated. Eyes small, finely faceted, interocular distance wide. Terminal maxillary palpomere more than three times as wide as long. Ligula narrow, the middle of front margin slightly emarginated. Labial palpus short, with the terminal palpomere semi-circular. Mentum slightly longer than width. Anterior margin of pronotum with translucent membranous areas, lateral and basal margins with narrow and complete marginal border. Well-marked punctations on anterior and posterior angles. Prosternum flat, narrowed between procoxal cavities, then broadening behind the coxa. Metaventrite process narrow. All the coxal lines absent.

Compared to female, male has longer antennae, stronger legs and wider tarsi. In some species, inner edge of the male femur has two small rows of nodules or odontoid processes.

### Rhodotritoma manipurica

Arrow, 1925

AF4A8730-1443-5E1C-A6DF-773A2CBAD281

*Rhodotritomamanipurica* Arrow, 1925 - Arrow 1925: 118

#### Materials

- **Type status:** Holotype. **Occurrence:** recordedBy: W. Doherty; individualCount: 1; **Location:** country: India; verbatimLocality: Assam, Manipur; **Identification:** identifiedBy: G.J. Arrow; **Record Level:** institutionCode: NHML
- **Type status:** Other material. **Occurrence:** recordedBy: Liang Tang; individualCount: 10; sex: 8 males, 2 females; **Location:** country: China; stateProvince: Tibet Autonomous Region; county: Milin County [米林县]; verbatimCoordinates: 29.2184°N, 94.1849°E; **Event:** year: 2005; month: 8
- **Type status:** Other material. **Occurrence:** recordedBy: Xiujuan Yang; individualCount: 1; sex: 1 female; **Location:** country: China; stateProvince: Yunnan; county: Lushui County, Pianma Town [泸水县，片马镇]; verbatimCoordinates: 26.0078°N, 98.6146°E; **Event:** year: 2004; month: 5

#### Description

**Figure 1.**
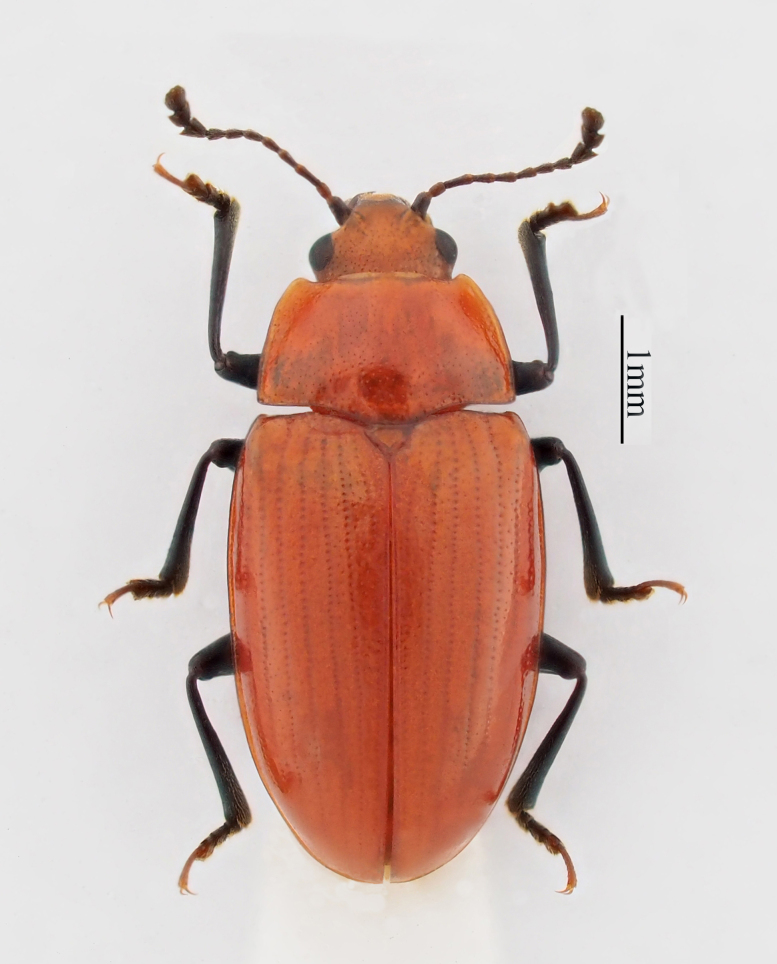
*Rhodotritomamanipurica* Arrow, 1925. Specimen from Tibet, China.

**Figure 2.**
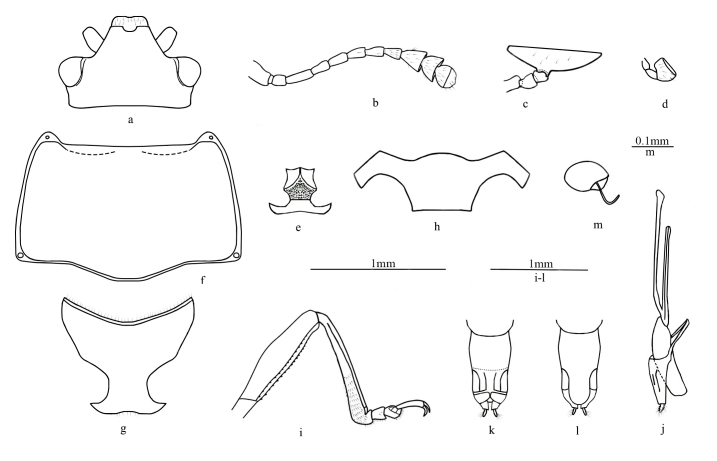
Morphological characters of *Rhodotritomamanipurica* Arrow, 1925. Scale bars: 1.0 mm or 0.1 mm. **a** head; **b** antenna; **c** maxillary palpus; **d** labial palpus; **e** mentum and submentum; **f** pronotum; **g** prosternum; **h** mesoventrite; **i** femur, protibia and protarsus; **j** aedeagus in lateral view; **k-l** female genitalia in ventral and dorsal views; **m** female spermatheca.

Body elongate-oval, convex dorsally, smooth and shining. General colour orange-yellow, the front edge of clypeus, legs and antenna black (Fig. 1). Head (Fig. 2a) small, with fine and sparse punctures. Clypeus without margin, anterior border emarginate. Frontoclypeal suture incomplete, presence on both sides. Compound eye small, prominent, finely faceted; interocular distance about 0.7 times width of head. Antenna (Fig. 2b) long and slender, exceeding the basal edge of pronotum, with golden setae; antennomere I rather large; antennomere III longer than others, antennomeres IV–VII nearly equal; antennomere Ⅷ slightly wider than VII, but significantly narrower than IX, antennomere Ⅸ triangular; antennomere X bowl-shaped; antennomere XI regularly rounded; relative lengths of antennomeres II–XI: 1.0: 1.6: 1.3: 1.2: 1.2: 1.2: 1.1: 1.4: 1.0: 1.4. Maxillary terminal palpomere (Fig. 2c) extremely wide, nearly 5 times as wide as long. Labial terminal palpomere (Fig. 2d) rubber hammer-shaped. Mentum (Fig. 2e) pentagon, middle area depressed; submentum small, trapezoidal.

Pronotum (Fig. 2f) nearly trapezoidal, widest at base (pronotum length/width ratio 0.7), slightly convex dorsally, with fine and dense punctate. Anterior border opposite head straight, margined behind eyes and sides; lateral border straight in basal half, converges slightly forward, strongly margined; basal border weakly sinuate, with narrow and complete margin, with coarse punctures on each side of sinuate. Anterior angle and posterior angle protruded and blunt, posterior angle almost rectangular. Scutellum pentagonal, with fine punctures, sharp posteriorly. Each elytron with nine distinct striae; intervals finely and sparsely punctate.

Prosternum (Fig. 2g) with textured surface; finely and sparsely punctate. Mesoventrite (Fig. 2h) broad, with coarse and sparse punctures. Metaventrite closely punctate at sides and sparsely punctate in middle.

Legs (Fig. 2i) slender; tibiae gradually widening to apex.

Male genitalia (Fig. 2j) with median lobe weakly curved; median strut straight and long, 2 times as long as median lobe. Female genitalia (Fig. 2k-l) with long styli at apex of coxite, covered with setae at apex. Female spermatheca (Fig. 2m) nearly kidney-shaped.

Body length: 4.5-6.0 mm; width: 2.0-4.0 mm.

#### Distribution

China (Tibet, Yunnan); India (Assam).

## Identification Keys

### Key to the species of *Rhodotritoma* from China

**Table:** 

1	Elytron bicolour	*R.albofasciata*
–	Elytron unicolour	2
2	Antennae uniform black	*R.manipurica*
–	Antennae biocolour	3
3	Metaventrite strongly and sparsely punctate	*R.coccinea*
–	Metaventrite finely and closely punctate	*R.rubicunda*

## Supplementary Material

XML Treatment for Rhodotritoma

XML Treatment for Rhodotritoma manipurica
